# A Daring Therapeutist

**Published:** 1878-04

**Authors:** 


A Daring Therapeutist.—At a late meeting of the Massachusetts Dental Society, Dr. Waters, of Salem, stated that bicarbonate of soda, such as used for cooking purposes, or any other alkali in neutral form, would afford instantaneous cessation of pain from the severest burns or scalds, and would cure such injuries in a few hours. Dipping a sponge into boiling water, the Doctor squeezed it over his right wrist, producing a severe scald around his arm and some two inches in width. Then, despite the suffering occasioned, he applied the scalding water to his wrist for half a minute. Bicarbonate of soda was at once dusted over the surface, a wet cloth applied, and the pain, the experimenter stated, was almost instantly deadened. Although the wound was of a nature to be open and painful for a considerable time, on the day following the single application of the soda the less injured portion was practically healed, only a slight discoloration of the flesh being perceptible. The severer wound, in a few days, with no other treatment than a wet cloth kept over it, showed every sign of rapid healing.—*Med. and Surgical Journal.*

